# A Morphometric Study of the Pars Plana of the Ciliary Body in Human Cadaver Eyes

**DOI:** 10.3390/vision8020030

**Published:** 2024-05-08

**Authors:** Jaime Guedes, Bruno F. Fernandes, Denisse J. Mora-Paez, Rodrigo Brazuna, Alexandre Batista da Costa Neto, Dillan Cunha Amaral, Adriano Cypriano Faneli, Ricardo Danilo Chagas Oliveira, Adroaldo de Alencar Costa Filho, Adalmir Morterá Dantas

**Affiliations:** 1 Department of Ophthalmology, Federal University of Rio de Janeiro, Rio de Janeiro 21941-971, RJ, Brazil; adroaldoalencar@hotmail.com (A.d.A.C.F.);; 2 Glaucoma Research Center, Wills Eye Hospital, Philadelphia, PA 19107, USA; dpaez@willseye.org; 3 Ophthalmology, Opty Group, Rio de Janeiro 22640-100, RJ, Brazil; rdchagas@gmail.com; 4 Argumento Institute, Boucherville, QC J4B-2G6, Canada; bruno.mtl@gmail.com; 5 Department of Ophthalmology, Federal University of the State of Rio de Janeiro, Rio de Janeiro 22290-240, RJ, Brazil; rdgbrazuna@gmail.com (R.B.); alexandrebcneto@gmail.com (A.B.d.C.N.); 6 Bahiana School of Medicine and Public Health, Salvador 40290-000, BA, Brazil; adrianofaneli@gmail.com; 7 Department of Ophthalmology, Federal University of Bahia, Salvador 40170-110, BA, Brazil

**Keywords:** pars plana, morphometry, anatomy

## Abstract

This study aimed to determine the pars plana length in postmortem human eyes using advanced morphometric techniques and correlate demographics to ocular metrics such as age, sex, ethnicity, and axial length. Between February and July 2005, we conducted a cross-sectional observational study on 46 human cadaver eyes deemed unsuitable for transplant by the SBO Eye Bank. The morphometric analysis was performed on projected images using a surgical microscope and a video-microscopy system with a 20.5:1 correction factor. The pars plana length was measured three times per quadrant, with the final value being the mean of these measurements. Of the 46 eyes collected, 9 were unsuitable for the study due to technical constraints in conducting intraocular measurements. Overall, the average axial length was 25.20 mm. The average pars plana length was 3.8 mm in all quadrants, with no measurements below 2.8 mm or above 4.9 mm. There were no statistically significant variations across quadrants or with age, sex, axial length, or laterality. Accurately defining the pars plana dimensions is crucial for safely accessing the posterior segment of the eye and minimizing complications during intraocular procedures, such as intravitreal injections and vitreoretinal surgeries.

## 1. Introduction

The ciliary body is a ring of tissue with dimensions typically ranging between 5–6 mm in width, and exhibits a triangular profile in cross-sectional views. Oriented with its base toward the anterior chamber and the apex merging with the anterior choroid posteriorly, the ciliary body is divided into two distinct regions: an anterior pars plicata (corona ciliaris) and a posterior pars plana (orbiculum ciliaris). The pars plicata is 2 mm wide and consists of 70 ciliary processes, each approximately 0.5–0.8 mm high and 0.5 mm wide. The pars plana is approximately 4 mm wide, from the posterior limits of the ciliary processes to the ora serrata, a serrated junction where the non-pigmented ciliary epithelium becomes the peripheral neural retina [1]. The pars plicata has three principal functions: accommodation, secreting the aqueous humor, and producing the fibers for the lens zonules, as well as the vitreous body’s glycosaminoglycans and collagen. There is also evidence that contraction of the ciliary muscle fibers distends the spaces within the trabecular meshwork, increasing the aqueous outflows and reducing the intraocular pressure [1]. The pars plana is mostly flat and pigmented, contains no ciliary processes, and consists primarily of muscle fibers and an inner epithelium layer [2]. Because the pars plana does not have a sensory function as the retina, nor is it involved in the accommodation or production of the aqueous or vitreous humor [1,3], it has long been recognized as a convenient anatomical landmark for interventions, including intravitreal injections, placement of intraocular slow-release implants, and introduction of surgical equipment during retinal surgeries [4,5,6]. Knowledge of the topographic anatomy of the pars plana and its boundaries avoids damage to the peripheral retina when accessing the vitreous cavity [7].

Since the early 20th century, the histological examination of cells, whether healthy or pathological, has been pivotal for the diagnosis and prognostication of diseases. Morphometry, a two-dimensional quantitative approach, is utilized for measuring dimensions such as length, perimeter, or area of anatomical structures to aid in diagnosis and to delineate tissue alterations in disease states. The objectives of employing morphometry are multifold: it reduces variability in cell and tissue feature quantification, supplies a reproducible and numerical scale for quantitative attributes, enhances the detection of subtle alterations, assesses the impact of varied tissue processing techniques, serves as a quality assurance mechanism, establishes shape and size benchmarks for educational and diagnostic purposes, functions as a valuable research instrument, and leverages the advancements in computational speed, cost-efficiency, and sophisticated data analysis algorithms [8,9]. Several areas of medicine, veterinary medicine, and dentistry, mainly pathology and anatomy, use morphometry for scientific research [10,11,12,13]. Morphometry is also used in ophthalmology, both in human and veterinary medicine [14,15,16]. The modern morphometry technique consists of microscopic images projected with the aid of a projector and using an object micrometer as a graduated ruler. The projection of this graduated ruler forms an enlarged image of the graduated scale. From there, with the aid of a centimeter ruler, the projection of the object micrometer is measured, establishing the conversion between the projection and the metric system [17]. Techniques in morphometry are classified based on the degree of automation and user involvement into four distinct types: manual, semi-automated, automated with user intervention, and fully automated [9].

The literature describes the anteroposterior length of the pars plana as 3.5 mm to 4.5 mm, depending on the study [8,10,11,18,19,20,21]. The differences can be explained by using different methodologies and instruments for measurements, including either fixed or fresh eyes, and differences in the demographic characteristics of the sample. Fixatives, such as formalin, dehydrate tissues that can alter the dimensions of intraocular structures, influencing the results. Some reports do not specify which quadrant was used for measurements, which is relevant since other studies have shown that the temporal pars plana is generally larger [18,20,22]. Lastly, differences in the average age of the study population might also explain the variability in measurements, as it is known that the pars plana is not fully developed at birth, and its length changes during the first years of life [4]. Right after birth, the ciliary body’s length is already significant. In infants aged 7 days to 6 months, the ciliary body averages 3.06 mm in length nasally (with a range from 2.60 to 3.45 mm) and 3.31 mm temporally (ranging from 2.80 to 4.30 mm), with the pars plana representing 73% to 75% of its total length. For infants aged 6 to 12 months, the lengths are 3.54 mm nasally (2.86 to 4.45 mm range) and 3.85 mm temporally (3.10 to 4.56 mm range). From 12 to 24 months, the average lengths are 3.87 mm nasally (3.28 to 4.48 mm range) and 4.14 mm temporally (3.46 to 4.99 mm range). The lengths continue to increase in children aged 24 to 72 months, reaching 4.28 mm nasally (3.75 to 4.95 mm range) and 4.94 mm temporally (4.15 to 5.50 mm range). These are compared to adult mean measurements of 4.79 mm nasally and 5.76 mm temporally, with the pars plana consistently making up 76% of the ciliary body’s total length [23].

The objective of this study was to accurately measure the length of pars plana in non-preserved human cadaver eyes using modern morphometric techniques and analyzing for potential correlations with age, gender, ethnicity, quadrants of the eye, and laterality.

## 2. Materials and Methods

### 2.1. Tissue Collection

A non-probabilistic sample of human cadaver eyes collected by the Eye Bank of the Brazilian Society of Ophthalmology (SBO) between February 2005 and July 2005 was analyzed in a cross-sectional observational study. All eyes studied were rejected for corneal or scleral transplantation according to the criteria adopted by the Eye Bank of the SBO. Donor-related data, such as medical record number, age, sex (male or female), ethnic group (white or non-white), and studied eye (right or left), were provided. Eyes that had suffered trauma at the site to be studied were excluded from the analysis. All eyes examined were from human cadavers whose legally responsible relatives provided written consent that the SBO Eye Bank and Rio Transplants could collect the organs for transplants or research in case they were not suitable for transplantation. The project was approved by the Research Ethics Committee of the Clementino Fraga Filho University Hospital—Federal University of Rio de Janeiro (129/04).

### 2.2. Tissue Preparation

**Figure 1:**
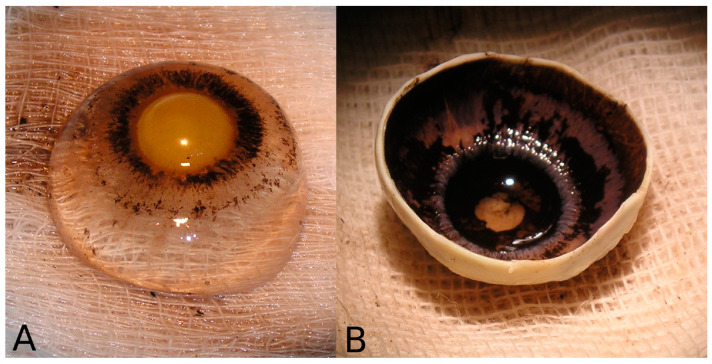
(**A**) Crystalline-vitreous complex removed after dissection. (**B**) Arrangement of the dissected eye for measurements.

After enucleation, the eyes were wrapped in surgical gauze, identified, and placed in an appropriate plastic container. The stump of the medial rectus tendon was left at a greater length than the other rectus muscles to facilitate the identification of the four quadrants: superior nasal (SN), inferior nasal (IN), superior temporal (ST), and inferior temporal (IT). The eyes were kept refrigerated between the telephone contact by the Eye Bank and the morphometric study, which ranged from 12 to 24 h. The axial length of the eyeball was measured using a 0.01 cm precision caliper. After identifying and marking the quadrants, the eyes were carefully dissected by making a coronal cut through an incision in the equatorial region using a scalpel and completed with scissors. Next, the anterior half was placed with the cornea facing downward, and the vitreous and crystalline lenses were removed so that the pars plana, the ciliary body, and the posterior face of the iris could be visualized (Figure 1).

### 2.3. Morphometry

Modern morphometry was used with the structures projected via a surgical microscope with a 12× magnification coupled with a video camera connected to a 14-inch television screen. The correction factor for the video-microscopy system was 20.5:1 (20.5 linear cm on the monitor was equivalent to 1.0 linear cm on the object) [9,17]. All measurements were performed following a single standardization in the same system, under the same microscope, at the same magnification, and in the same place (Hospital Universitário Clementino Fraga Filho, Federal University of Rio de Janeiro, Brazil). Measurements of the pars plana were corrected to account for the curvature of the eye using the Littmann method, ensuring an accurate representation of the three-dimensional structure. The length of the pars plana was measured from the posterior limit of the ciliary processes (pars plicata) to the ora serrata in triplicates for each quadrant. The final value for each quadrant was the result of the arithmetic mean of the three measurements.

### 2.4. Statistical Analysis

The mean, median, and standard deviation (SD) were calculated for the axial length and the measurements of the pars plana in all quadrants using an Excel^®^ data sheet. Statistical analysis assessed the impact of different variables: laterality, sex, ethnicity, and age. Since the mean and median values were similar, indicating a symmetrical sample close to a sample with a normal distribution, the differences between the means of the variables were studied using the Student’s *t*-test. The criterion for determining significance was a level of 5%; that is, when the *p*-value of the statistical test was less than or equal to 0.05, it was considered statistically significant. To compare attributes related to a variable between two groups (e.g., the right and left eyes), the Wilcoxon non-parametric test was used with a confidence level of 95%, which was more appropriate for the sample size. Pearson’s correlation coefficient (r) was used to analyze the correlation between attributes, with a confidence level of 95%.

## 3. Results

### 3.1. Demographics

From a total of 46 eyes of 32 donors, statistical analyses were performed on a sample with 37 eyes from 28 donors. The final analysis did not include nine eyes because of the technical inability to obtain all intraocular measurements. Twenty (54%) of the studied eyes were left eyes, 26 (70%) were from white donors, 27 (73%) were from men, and 36 (97%) were from adult donors (18 years or older). The mean (SD) age of the donors (*n* = 28) was 53.85 (19.17) years, with a median (range) of 56 (6–88) years.

### 3.2. Morphometry

Table 1 shows the summary of the axial length and the length of the pars plana in all quadrants for the 37 eyes included in the statistical analysis. The mean (SD) axial length of the only child was 27.00 (1.25) mm, and for the eyes of adult donors (*n* = 36), it was 25.16 (0.67) mm. Subgroup analysis showed a mean axial length of 25.20 (0.9) mm in females (*n* = 10), 25.20 (0.6) mm in males (*n* = 27), with an SD of 0.60 mm, 25.20 (0.80) mm in whites (*n* = 26), and 25.30 (0.6) mm in non-whites (*n* = 11). The mean length of the pars plana was 3.8 mm in all quadrants. The widest variation was seen in the superior temporal quadrant, with a 2.8–4.9 mm range, and the narrowest range in the inferior temporal quadrant (3.1–4.5 mm). There was no measurement in any quadrant of any eye smaller than 2.8 mm or larger than 4.9 mm (both measurements were seen in the superior temporal quadrant).

Student’s *t*-tests showed no significant differences concerning sex or ethnicity and the length of pars plana in any quadrant (Table 2). Pearson’s correlation test results (r) with a confidence level of 95% showed no linear relationship between the size of the pars plana in any quadrant with axial length (SN: −0.111, *p* = 0.511; IN: −0.177, *p* = 0.295; ST: −0.127, *p* = 0.453; IT: −0.106, *p* = 0.533) or age (SP: 0.187, *p* = 0.267; IN: 0.155, *p* = 0.360; ST: 0.230, *p* = 0.170; IT: 0.146, *p* = 0.390). The Wilcoxon non-parametric test with a confidence level of 95% showed no significant differences in the size of the pars plana between the right and left eyes in any quadrant.

## 4. Discussion

In the present study, we examined the dimensions of the pars plana across all four quadrants in 37 eyes, with an emphasis on identifying potential variations linked to demographic factors such as sex and ethnicity, as well as biological parameters like axial length and age. Our findings revealed a uniform mean pars plana length of 3.8 mm across all quadrants, within the previously reported size range of 3.5 mm to 4.5 mm [18,19,20,22,24,25,26,27]. This mean is slightly below the range reported by Saraux et al. [20] and below the average found by Forrester et al. [1], yet it aligns with the findings of Duke-Elder [22]. The widest variability was observed in the superior temporal quadrant, ranging between 2.8 mm and 4.9 mm. Conversely, the inferior temporal quadrant exhibited the narrowest variation. Notably, the statistical analyses, which included Student’s *t*-tests and Pearson’s correlation, alongside the Wilcoxon non-parametric test, consistently indicated no significant differences or correlations between the measured lengths of the pars plana and the aforementioned demographic or biological factors.

Most prior research utilized methodologies that precede contemporary morphometric advancements. In most of the studies cited in classic books regarding dimensions of the pars plana, measurements were taken before current morphometry techniques were available. At the time of some of these studies, there were few specific instruments for the measurement or suitable means of conservation, microscopes were less advanced, and measuring intraocular structures through on-screen projections was not possible. Saraux et al. described the pars plana as having a 4–4.5 mm length [20], without mentioning the differences between the temporal and nasal sides [20]. Forrester et al. reported a length of 4 mm, again without citing the differences between the temporal and nasal sides [1]. Duke-Elder found that the length of the pars plana ranged from 3.6 to 4 mm [22]. Lastly, Dantas described the pars plana length ranging from 4.5 mm on the temporal horizontal meridian to 3.5 mm on the nasal side [18].

In 1989, Bonomo published an anatomical-topographical study of the pars plana, pars plicata, and ora serrata in 15 eyes of fetuses obtained from autopsies [4]. The mean value of pars plana size was 1.15 ± 0.32 mm in the nasal, 1.20 ± 0.34 mm in the temporal, 1.19 ± 0.34 in the superior meridian, and 1.15 ± 0.34 mm in the inferior meridians. Contrary to the results presented herein, there was a significant correlation with the anteroposterior diameter, and significant differences between the mean sizes of the pars plana in the superior meridian were found compared to the nasal and inferior meridian. Aiello et al. investigated the postnatal development of the ciliary body and pars plana in 76 normal eyes of subjects from 1 week to 6 years of age. The study showed that the pars plana grow rapidly during the first years of life, from 2.23 ± 0.06 and 2.48 ± 0.07 mm before six months of age to 3.25 ± 0.11 and 3.85 ± 0.12 at 24–78 months until reaching the adult dimensions of 3.64 ± 0.11 and 4.32 ± 0.13 mm and in the nasal and temporal meridians, respectively [23]. Although we did not find the differences between meridians in our study, the largest variability and dimensions were indeed found in the temporal quadrant. Of note, in the Bonobo study, the eyes were fixated in 50% ethanol for 2–6 days before measurement, and in Aiello et al., measurements were performed on formalin-fixed, paraffin-embedded stained sections under a microscope. In contrast, our samples were sent directly from the SBO Eye Bank for measuring without any preserving agent that can dehydrate tissue and alter its dimensions.

Dividing the eye into quadrants for this morphometric study is supported by literature indicating variability in the pars plana lengths between nasal and temporal regions, with the temporal typically being larger [23]. Beyond comparing hemispheres, quadrant measurement offers a more comprehensive assessment [18,20,22]. Contrary to the literature, no significant differences were seen in this study among mean measurements in the four quadrants, even though the greatest dimensions were still found in the temporal quadrants (4.9 mm), consistent with Dantas’ findings [18]. We believe some outlier readings as low as 2.8 mm in the superior temporal quadrant might have skewed the results. While larger sample sizes could mitigate the influence of individual variability, it is essential to note that this variability exists for proper intraocular procedure planning. In some eyes, the length of the pars plana can be significantly shorter than the accepted average range of 3.5–4.5 mm [18,19,20,22,24,25,26,27]. The consequence of miscalculating the length of the pars plana is accessing the vitreous cavity posterior to the ora serrata, with the potential of damaging the peripheral sensory retina leading to vision-threatening complications, including retinal detachment and vitreous hemorrhages.

In our study, no statistically significant differences were observed between white participants and those from other ethnicities. This binary categorization was employed due to the complexity of defining ethnic groups within the Brazilian population and the limitations in the ethnic data found in the medical records. Consistent with existing literature, no significant differences were noted between genders or between right and left eyes [7,18,20,22]. However, contrary to previous findings, our results did not indicate a correlation between the length of the pars plana and axial length [28]. One study on 450 enucleated adult eyes found the pars plana length significantly shorter in hyperopes with an axial length of 21.5–22.9 mm compared to high myopes with an axial length of over 24.8 mm [29]. We hypothesize that the reason we did not find such an association between pars plana and axial length is the relatively limited range (23.5–27 mm) of axial lengths found in our sample. Another study using in vivo imaging of the scleral shadows of pars plana demonstrated that in patients with axial lengths ranging from 20–22.9 mm, 23–24.9 mm, and >25 mm, the pars plana width was 3.4 mm, 4.16 mm, and 4.95 mm, respectively [28]. It is important to note that in vivo measurements of the axial length rely on different anatomical landmarks, thus yielding different results than measurements in enucleated eyes. In our study, the axial length was defined as the distance between the anterior apex of the cornea and the posterior side of the sclera. Axial length measurement via A-scan involves calculating the span from the anterior surface of the cornea to the retina’s inner limiting membrane [30]. This method often produces underestimations of axial length, which may stem from the compression of the cornea caused by the transducer’s direct contact, leading to decreased corneal thickness or anterior chamber depth [31,32]. Alternatively, optical biometry employs optical partial coherence interferometry (PCI) to determine the axial length in vivo, quantifying the distance from the cornea’s anterior surface to the retinal pigment epithelium [30]. Understanding the pars plana is crucial for various procedures, including cyclophotocoagulation and trabeculectomy, to ensure effective and safe treatment outcomes [33,34].

The current study has some limitations that warrant consideration. The small cohort of children’s eyes—only one of six collected eyes was included—restricts our ability to compare anatomical differences between adult and pediatric populations. This difficulty in delineating anatomical features in younger eyes has also been reported by others, such as Bonomo et al. [4], who noted incomplete development in these regions in children. Thus, only one child’s eye was assessed in our study. Further research with a more substantial pediatric sample is required to ascertain developmental differences. Second, the diverse ethnic composition of the Brazilian population poses a challenge in defining distinct ethnic categories. Also, the regional constraints of our sample size require caution when extending these findings to populations beyond Brazil. Third, despite a power analysis affirming the adequacy of our sample size for statistical evaluation, the total number of 37 eyes from 28 donors remains relatively small, raising the possibility that our results could be influenced by random variation. Future studies would benefit from a larger sample to mitigate this risk. Lastly, the pars plana was measured on a two-dimensional picture of the inner side of the dissected front part of the eyeball, without considering the eye’s curvature. Although the distances are rather small for such artifacts to have a meaningful impact on our study, it is possible that a different technique measuring the pars plana along the curvature of the globe could yield different results.

The accurate delineation of the pars plana dimensions is paramount due to its role as an access for a range of ophthalmic surgical procedures, such as vitrectomy, the delivery of drugs into the vitreous cavity, and the placement of various implants. Our investigation, leveraging modern morphometric methodologies, has demonstrated variations in the size of the pars plana, with some instances revealing a length that is less than what has been previously recorded in the medical literature. These observations are crucial as they aid ophthalmic surgeons in tailoring their surgical techniques and provide a reliable anatomical benchmark for further scholarly inquiry. Future research, potentially employing probabilistic models and advanced morphometric analysis, can benefit from the data collected in our study. By embracing these modern approaches to investigate the pars plana, we can expect to see refinements in surgical methods that are more closely aligned with the anatomic realities presented by individual patients. Ultimately, the implications of our findings aim to advance surgical precision and, consequently, improve clinical outcomes for those undergoing eye surgeries. It is this nexus of innovative research and practical surgical application that our study contributes to, paving the way for improved patient care in ophthalmology.

## Figures and Tables

**Table 1:** Morphometry of the axial and pars plana length in non-preserved human cadaver eyes.

	Average (Standard Deviation)	Median (Range)
Axial length (mm)	25.2 (0.73)	25 (23.5–27)
Pars plana length (mm)		
Superior nasal	3.8 (0.4)	3.9 (3.0–4.6)
Inferior nasal	3.8 (0.3)	3.8 (3.1–4.5)
Superior temporal	3.8 (0.5)	3.9 (2.8–4.9)
Inferior temporal	3.8 (0.3)	3.8 (3.0–4.9)

**Table 2:** Statistical results for the axial and pars plana length in all quadrants in relation to sex and ethnicity.

		Axial Length, Average (SD) in mm	SN, Average (SD) in mm	IN, Average (SD) in mm	ST, Average (SD) in mm	IT, Average (SD) in mm
**Sex**	Men (*n* = 27)	25.2 (0.6)	3.83 (0.48)	3.79 (0.39)	3.79 (0.53)	3.85 (0.44)
	Women (*n* = 10)	25.2 (0.9)	3.87 (0.45)	3.86 (0.44)	4 (0.52)	3.96 (0.42)
	*p*-value *	>0.05	>0.05	>0.05	>0.05	>0.05
**Ethnicity**	White (*n* = 26)	25.2 (0.8)	3.86 (0.43)	3.84 (0.35)	3.91 (0.45)	3.9 (0.3)
	Non-white (*n* = 11)	25.3 (0.6)	3.79 (0.42)	3.72 (0.39)	3.71 (0.41)	3.82 (0.26)
	*p*-value *	>0.05	>0.05	>0.05	>0.05	>0.05

* *p*-value obtained using the Student’s *t*-test. SN: superior nasal; IN: inferior nasal; ST: superior temporal; IT: inferior temporal, SD: standard deviation.

## Data Availability

Data are contained within the article.
